# The effect of NSAIDs on postfracture bone healing: a meta-analysis of randomized controlled trials

**DOI:** 10.1097/OI9.0000000000000092

**Published:** 2021-03-22

**Authors:** Humaid Al Farii, Leila Farahdel, Abbey Frazer, Ali Salimi, Mitchell Bernstein

**Affiliations:** Division of Orthopaedic Surgery, McGill University, Montreal, Canada

**Keywords:** fracture, nonunion, NSAIDs

## Abstract

To determine whether nonsteroidal anti-inflammatory drugs (NSAIDs) have an adverse effect on bone healing by evaluating all available human randomized controlled trials (RCTs) on this subject.

**Data Sources:**

A comprehensive search of electronic databases (PubMed, MEDLINE, and Cross-References) until October 2018 comparing the occurrence of nonunion in patients who received NSAIDs to the control group through RCTs.

**Study Selection:**

Inclusion criteria were English-only studies, and the type of studies was restricted to RCTs.

**Data Extraction:**

Two authors independently extracted data from the selected studies, and the data collected were compared to verify agreement.

**Data Synthesis:**

Nonunion was the main outcome evaluated in each study. Regression analysis was used to estimate the relative risk comparing the duration and the type of NSAIDs by calculating the odds ratio (OR) for dichotomous variables. Studies were weighed by the inverse of the variance of the outcome, and a fixed-effects model was used for all analyses.

**Conclusions:**

Six RCTs (609 patients) were included. The risk of nonunion was higher in the patients who were given NSAIDs after the fracture with an OR of 3.47. However, once the studies were categorized into the duration of treatment with NSAIDs, those who received NSAIDs for a short period (<2 weeks) did not show any significant risk of nonunion compared to those who received NSAIDs for a long period (>4 weeks). Indomethacin was associated with a significant higher nonunion rate and OR ranging from 1.66 to 9.03 compared with other NSAIDs that did not show a significant nonunion risk.

## 1 Introduction

Tissue damage triggers the processes of coagulation, inflammation, and healing. The healing process for soft tissue around bone is defined by regeneration and repair that matures into scar tissue, whereas the bone tissue itself has a unique ability to regenerate shape, strength, and preinjury function. Fracture healing is a combination of a complex and sequential set of events that depends on the stability of the fracture.^[1,2]^

There are many factors that influence the healing process, including environmental factors, drugs, and physiological changes. One of these factors is the use of NSAIDs, which is commonly used in patients as an anti-inflammatory as well as an analgesic. NSAIDs effect on bone healing has been studied for many years, with results showing debatable effects on bone healing.

There have been multiple studies published evaluating the benefits and consequences of NSAIDs in bone healing of both animal and human tissue in vitro and in vivo. The studies that showed that NSAIDs have a significant effect on bone healing are categorized by mechanism of action of NSAIDs in Table 1.^[3–34]^

**Table 1:** Animal studies which showed effect on bone healing with the use of NSAIDs.

Drug classification	NSAID	Duration	References
Acetic acid derivatives	Indomethacin	Long duration	Bo et al (1976)^[3]^
			Sudmann et al (1979)^[4]^
			Allen et al (1980)^[5]^
			Tornkvist et. al (1984)^[6]^
			Sato et al (1986)^[7]^
			Keller et al (1989)^[8]^
			Hogevold et al (1992)^[9]^
			Engesaeter et al (1992)^[10]^
			Altman et al (1995)^[11]^
			Dimar et al (1996)^[13]^
			Long et al (2002)^[14]^
			Riew et al (2003)^[15]^
			Persson et al (2005)^[16]^
			Karachalios et al (2007)^[17]^
	Indomethacin	Short duration	Reikeraas et al (1998)^[12]^
	Ketorolac	Long duration	Ho et al (1998)^[18]^
			Gerstenfeld et al (2007)^[19]^
	Diclofenac	Long duration	Beck et al (2003)^[20]^
			Sen et al (2007)^[21]^
			Bissinger et al (2016)^[22]^
	Etodolac	Short duration	Endo et al (2005)^[23]^
Enolic acid derivatives	Tenoxicam	Short duration	Giordano et al (2003)^[24]^
			Sen et al (2007)^[21]^
	Meloxicam	Long duration	Ribeiro et al (2006)^[25]^
			Karachalios et al (2007)^[17]^
Propionic acid derivatives	Ibuprofen	Long duration	Tornkvist et. al (1984)^[6]^
			Obeid et al (1992)^[26]^
			Leonelli et al (2006)^[27]^
			O’Connor et al (2009)^[28]^
	Naproxen	Long duration	Goodman et al (2006)^[29]^
			Kaygusuz et al (2006)^[30]^
Salicylates	Aspirin	Long duration	Allen et al (1980)^[5]^
Selective COX-2 inhibitors	Parecoxib	Long duration	Gernstenfeld et al (2003)^[31]^
	Rofecoxib	Long duration	Goodman et al (2006)^[29]^
			Leonelli et al (2006)^[27]^
			Murnaghan et al (2006)^[32]^
			Karachalios et al (2007)^[17]^
			O’Connor et al (2009)^[28]^
	Celecoxib	Short duration	Bergenstock et al (2005)^[33]^
			Simon and O’Connor (2007)^[34]^
			Long et al (2002)^[14]^
	Celecoxib	Long duration	Simon and O’Connor (2007)^[34]^
	Valdecoxib	Long duration	Gerstenfeld et al (2007)^[19]^

COX-2 = cyclooxygenase-2.

There have been multiple studies published evaluating the benefits and consequences of NSAIDs in bone healing of both animal and human tissue to clinical RCTs. Subsequently, there are animal studies that showed NSAIDs do not have a significant effect on bone healing, which are listed in Table 2.^[35–49]^ In conjunction to several RCTs, there has been meta-analysis of strong animal studies, yet the effect of NSAIDs on bone healing is controversial and many surgeons avoid these medications because of the concern of the possible delay in healing.^[13,50–52]^

**Table 2:** Animal studies which showed no effect on bone healing with the use of NSAIDs.

Drug classification	NSAIDs	Duration	References
Acetic acid derivatives	Indomethacin		Elves et al (1982)^[35]^
			Sudmann et al (1982)^[36]^
			Boiskin et al (1988)^[37]^
			Mbugua et al (1989)^[38]^
			Keller et al (1990)^[39]^
			Brown et al (2004)^[40]^
	Ketorolac	Long duration	Mullis et al (2006)^[41]^
	Ketorolac	Short duration	Fracon et al (2010)^[42]^
	Diclofenac	Long duration	Tiseo et al (2006)^[43]^
Enolic acid derivatives	Meloxicam	Long duration	Van de Heide et al (2008)^[44]^
Propionic acid derivatives	Ibuprofen	Long duration	Tornkvist et al (1980)^[45]^
			Huo et al (1991)^[46]^
			Mullis et al (2006)^[41]^
	Ketoprophen	Long duration	Urrutia et al (2007)^[47]^
			Van de Heide et al (2008)^[44]^
Selective COX-2 inhibitors	Celecoxib	Long duration	Brown et al (2004)^[40]^
			Mullis et al (2006)^[41]^
	Rofecoxib	Long duration	Mullis et al (2006)^[41]^
			Tiseo et al (2006)^[43]^
			Hak et al (2011)^[48]^
	Etoricoxib	Short duration	Fracon et al (2010)^[42]^
Sulfonanilides	Nimesulide	Short duration	Teofilo et al (2011)^[49]^

COX-2 = cyclooxygenase-2.

The purpose of a meta-analysis of only randomized control human trials is to provide a greater understanding of the true effect of NSAIDs on human bone healing, and the risk of nonunion. Secondary outcome measures, duration of NSAIDs use, and type of NSAIDs will also be included to determine its effect on healing complications.

## 2 Material and methods

This meta-analysis was conducted following the Preferred Reporting Items for Systemic Reviews and Meta-Analyses Statement. The study was deemed exempt from Institutional Review Board and Animal Use Committee Review. Informed consent was not applicable in our study. A comprehensive meta-analysis of the available clinical evidence was performed, with regards to NSAIDs exposure and nonunion risk in humans. Studies were excluded if long bone fractures were not studied, such as dental and spine.

**Figure 1:**
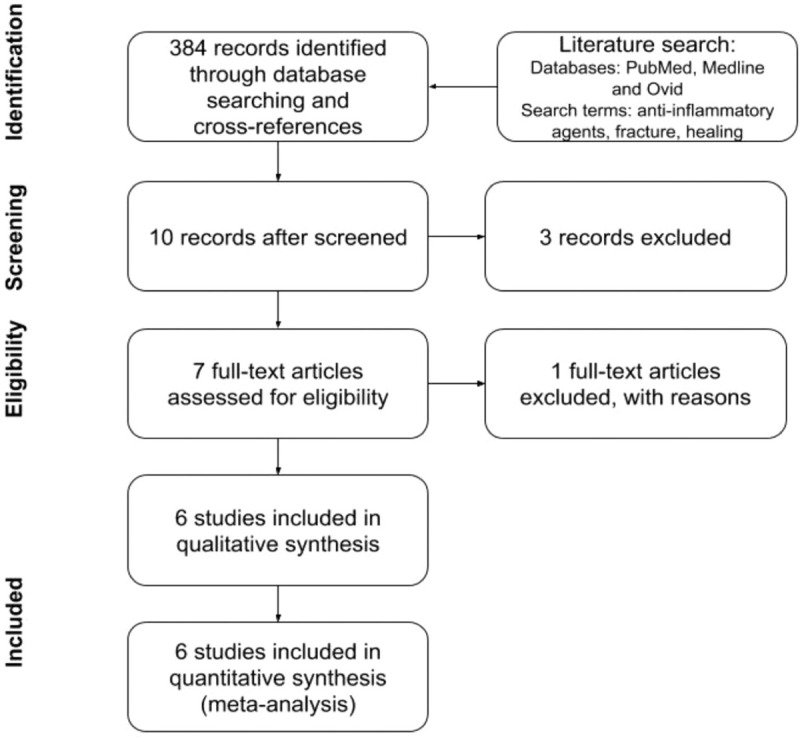
Flow chart illustrating the review process.

A literature search was conducted through PubMed and Medline databases and restricted to English literature. The search terms used were “anti-inflammatory agents,” “fracture,” “healing,” with expanded search terms with Boolean operators being used. The period of search included any studies until October 2018. Manual searching of studies was also done through review articles and other relevant material, through the snowballing technique. Gender and age group were not limited. Neither the type of NSAIDs nor the duration postoperatively was used as a restriction to the study. The type of study was restricted to human RCTs only. The summary of the study flow is provided in Figure 1.

Two reviewers reviewed each title independently and only relevant abstracts were included after the screen, which included 10 studies. From these studies, 3 were excluded. One was a study on regenerative periodontal treatment for which the healing process is different from bone healing and was, therefore, excluded.

The data extraction from each study includes year of publication, randomization method, patient, and treatment characteristics. The studies were evaluated specifically for publication bias using a funnel plot. RevMan software (version 5.3, The Cochrane Collaboration) was used for the analysis. Treatment effects were estimated by calculating the OR with 95% confidence interval (CI) for dichotomous variables. Studies were weighed by the inverse of the variance of the outcome, and a fixed-effects model was used for all analyses.

## 3 Results

A total of 384 studies were initially screened, where 10 studies were considered for inclusion. From those, 2 were excluded because they discussed pain management post spinal fusions, and a third study on healing of enamel matrix, was excluded because the healing process differs from long bones. Additionally, the study that included the results from Study to Prospectively Evaluate Reamed Intramedullary Nails in Patients with Tibial Fractures trial described qualitative results; however, it did not provide quantitative information on their conclusions on NSAIDs use for bone healing. A total of 6 randomized control studies were considered for final analysis (Table 3).^[53–58]^ The data from these 6 studies were extracted for analysis.

**Table 3:** Summary of human RCTs on NSAIDs effect on bone healing, used in this meta-analysis.

Study	No.	Bone type and management	NSAID	Length of exposure	Dose	Nonunion diagnosis	Conclusion
Adolphson et al (1993)^[53]^	42	Displaced Colles fracture Closed reduction	Piroxicam RD: Paracetamol	8 weeks	20 mg /d	Xray	No difference in radial shortening Small but no significant reduction in osteopenia in the piroxicam group after 8 weeks
Brattwall et al (2010)^[54]^	100	Elective hallux valgus surgery	COX-2 inhibitors RD: paracetamol, oxycodone	7 days	Etoricoxib 120 mg for 4 days then 90 mg for 3 days	CT scan + Clinical evaluation	None of the CT scans showed limited bone healing
Burd et al (2003)^[55]^	112	Prophylaxis post acetabular fracture ORIF + long bone fracture	Indomethacin	6 weeks	25 mg TID	X-ray	Risk of nonunion significant for indomethacin to control, 5.32 to 1
Davis et al (1988)^[56]^	100	Colles’ fractures	Flurbiprofen RD∗: paracetamol	14 days	50 mg x 3–6 daily	X-ray	No significant difference with placebo for anatomic position
Drendel et al (2009)^[57]^	336	Simple Arm fracture in pediatrics (radius, ulna, or humerus)	Ibuprofen	Variable <2 weeks	Avg. 4 doses of 10 mg/kg	Telephone follow-up and file review	No association between refracture or nonunion No direct endpoint related to bone healing
Sagi et al (2014)^[58]^	98	Acute acetabular fracture treated operatively	Indomethacin	3 days to 6 weeks	75 mg daily	CT scan	Tx 1 week of indomethacin may be beneficial for healing, without increase of nonunion Tx 6 weeks of indomethacin increases the incidence of nonunion

No. = number of patients, ORIF = open reduction internal fixation, RD = rescue drug; Tx, treatment.

### 3.1 Study characteristics and quality

**Figure 2:**
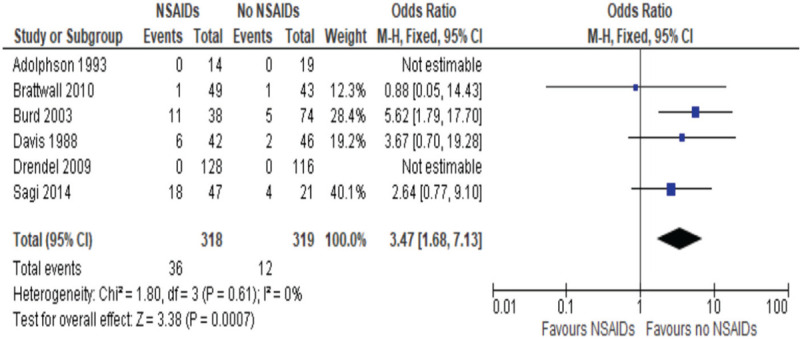
Odds ratios of risk of nonunion post fracture healing, NSAIDs vs non-NSAIDs use.

From all of the studies that were included, there were 609 patients included where 290 were exposed to an NSAID compared to 319 control patients. Included in these studies are Adolphson et al and Drendel et al, which had no events of nonunion in either arms of the studies.^[53,57]^ Based on the Cochrane handbook for meta-analysis, the standard practice for the calculations of OR and risk ratio is to exclude the studies where there are no events in the treatment group and in the control.^[59]^ In the calculation table, it can be seen that the studies are included; however, they do not have weight attributed to the total analysis on the 6 studies, as described in Figure 2. From these studies, 3 considered long bone fractures, Colles fracture,^[53,56]^ or simple arm fractures (radius, ulna, or humerus), 2 studies considered acetabular fractures,^[55,58]^ and 1 studied elective hallux valgus surgery.^[54]^

Each study was an RCT comparing NSAIDs with a control. Adolphson et al studied piroxicam 20 mg per day, Brattwall et al studied COX-2 inhibitors etoricoxib (120 mg for 4 days then 90 mg for 3 days) or tramadol (200 mg for 7 days),^[53,54]^ Davis and Ackroyd^[56]^ studied flurbiprofen 150 to 300 mg daily for 2 weeks, Drendel et al^[57]^ studied ibuprofen, whereas Burd et al and Sagi et al studied indomethacin from 3 days to 6 weeks.^[55,58]^

**Figure 3:**
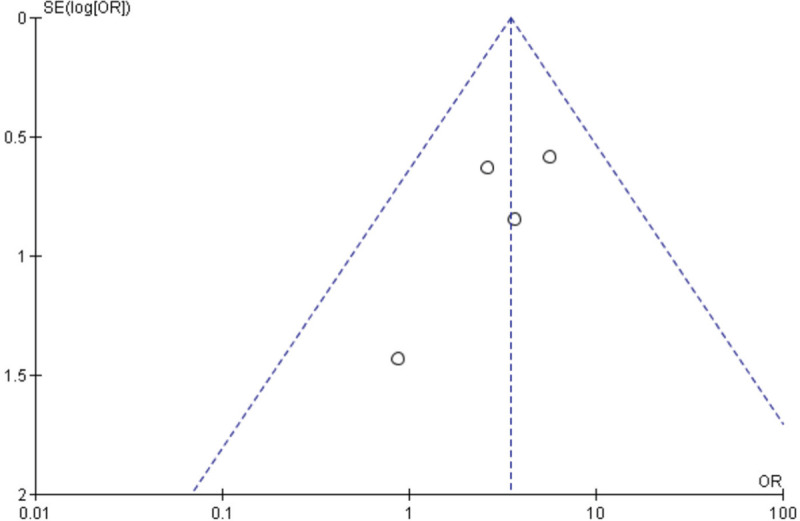
Funnel plot of the studies included in the meta-analysis.

The diagnosis of nonunion was made through either X-ray of the fracture and CT scan or telephone follow-up and file review. From the studies included, the ORs were well distributed in terms of publication bias (Fig. 3).

A power analysis on the selected studies and the subgroups was conducted to understand the limitation from having a limited number of cases per study and its significance. The power refers to the probability that the test will find a statistically significant difference when such a difference exists. To calculate this, a type I error level (alpha) of 0.05 was used. A type II error (beta) of 0.2 was used that corresponds to a power of 0.8. Based on a high heterogeneity group, with a minimum power of 0.8, ideally it would need 5 RCTs of 50 patients in each group, 10 RCTs of 30 patients in each group, or 15 RCTs of 20 patients in each group.

In the case of this meta-analysis, there are 4 RCTs with an average of 43 patients per group. Using these values, with an effect size of 0.5, this is considered to have a power of 100%, which means for large differences, this is a significant meta-analysis. However, with the effect size of 0.2, the power is 74.6%, which is less than 80%, which means for small differences, this number of studies is not sufficient to find significance.

### 3.2 General effect of NSAIDs on bone healing and formation of nonunion

From those studies, there were 48 nonunion (36 for NSAIDs vs 12 for non) , defined as a bone that fails to heal, of which 36 were exposed to NSAIDs and 12 were not. The 4 studies resulted in an OR of 3.47, with a 95% CI of 1.68 to 7.13. The significance level is *P* = .001, as described in Figure 2. This indicates that there is an increased risk of nonunion with NSAIDs exposure which is significant.

### 3.3 Effect of NSAIDs for short duration compared to long duration on bone healing

**Figure 4:**
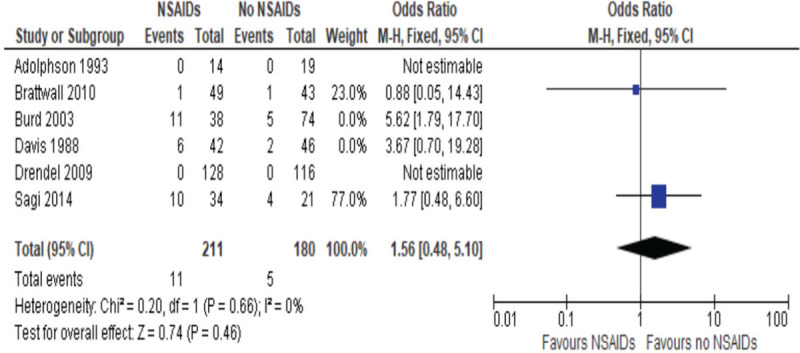
Odds ratios of risk of nonunion postfracture healing, NSAIDs vs non-NSAIDs short-term use (less than 2 weeks).

**Figure 5:**
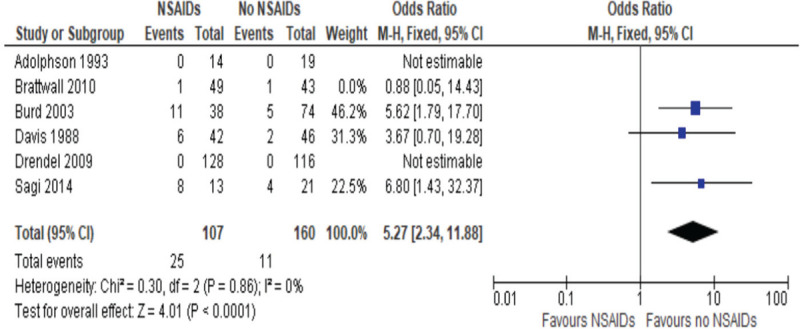
Odds ratio of risk of nonunion post fracture healing, NSAIDs compared to control long term use (greater than 2 weeks).

A subgroup analysis was performed classifying the duration of NSAIDs administration, with a short duration defined as less than 2 weeks and a long duration defined at more than 2 weeks. Short duration studies included 2 studies,^[54,57,58]^ with the OR of 1.56, 95% CI between 0.48 and 5.10, and *P* value of 0.484, as described in Figure 4, whereas the 4 studies included in the long duration, including 1 study, Sagi et al, which quantified both short and long duration,^[53,55,56,58]^, had an OR of 5.27, 95% CI between 2.34 and 11.88 and the *P* value of less than .0001, as described in Figure 5. This indicates that although short use of NSAIDs has no significant effect on bone healing, long duration of more than 2 weeks has a significant higher rate of nonunions.

### 3.4 Effect of indomethacin NSAIDs compared to other NSAIDs on bone healing

**Figure 6:**
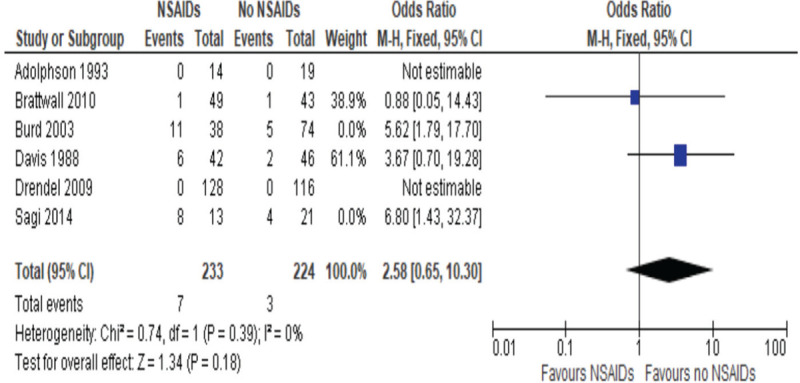
Odds ratio of risk of nonunion post fracture healing, NSAIDs not including indomethacin compared to no NSAIDs.

**Figure 7:**
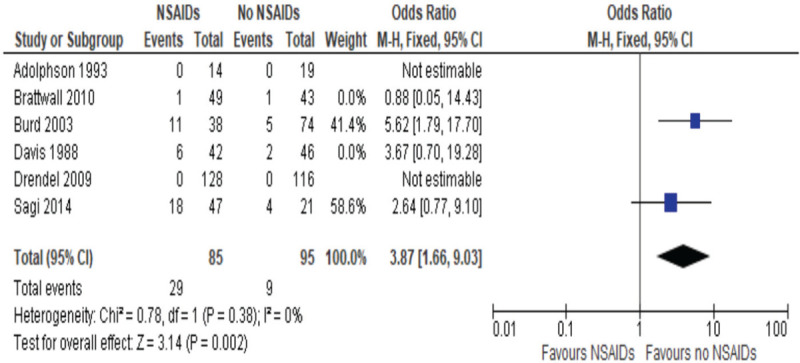
Odds ratio of nonunion with indomethacin use compared to no NSAIDs.

A subgroup analysis was performed classifying the NSAIDs with the exception of indomethacin. From the studies, 4 studies administered NSAIDs that were not indomethacin.^[53,54,56,57]^ The OR was found to be 2.58 with 95% CI 0.65 to 10.30, the *P* value of .18, therefore, it is not significant, as described in Figure 6. This means that NSAIDs that did not include indomethacin did not have a significant effect on nonunion, compared to studies that included only indomethacin as NSAIDs,^[55,58]^ had an OR of 3.87 with 95% CI 1.66 to 9.03, and a *P* value of .002, as described in Figure 7. This means that indomethacin used as an NSAID has a significant effect on healing leading to nonunions.

## 4 Discussion

NSAIDs are commonly used as part of pain management postfracture. The negative effect of NSAIDs is mostly related to the inhibition of cyclooxygenase (COX) enzyme activity and the prostanoid pathway. There are 2 forms of COX enzymes that have been isolated. COX-1 takes part in the synthesis of prostaglandins under normal physiological conditions, whereas COX-2 is synthesized under an inflammatory state and induces the release of prostaglandins. NSAIDs decrease the activity of COX isoenzymes that decrease the synthesis of prostanoids. Research by Simon et al states that COX-2 is necessary for normal endochondral ossification during fracture healing.^[60]^ NSAIDs are generally classified by chemical structure or mechanism of action. Some of the commonly used classes are salicylates, propionic acid derivatives, acetic acid derivates, enolic acid derivatives, and selective COX-2 inhibitors. It can be understood that there is a difference in effect on bone healing with the NSAIDs due to the variation of the mechanism of action of NSAIDs.

There have been about 50 animal studies that were published on the effect of NSAIDs showing a variety of results. These studies are described in Tables 1 and 2. The animal studies included NSAIDs from different classes of NSAIDs. Animal studies investigated various NSAIDs during short-term and long-term use while looking at end point factors including mechanical properties of bone, histological grade, and radiological appearance. From these studies, most had greater support that NSAIDs delay or prevent bone healing, whereas others denied that effect. However, the question remains if this is true in humans. A thorough meta-analysis of human studies done by Wheatly et al,^[61]^ which included pediatric and adult bone healing and was not limited to RCT studies, showed that NSAIDs exposure increased delayed union or nonunion. However, there is no significance in the pediatric population, or with low-dose or short-duration use.

The purpose of this meta-analysis was to analyze RCTs that studied the use of NSAIDs in bone healing. This meta-analysis of 6 RCTs evaluated 609 patients, of whom 290 were exposed to an NSAID compared to 310 control patients. From these studies, subcategories were further studied. When comparing all the included studies, there was a significant increased risk of nonunions when exposed to NSAIDs with an OR of 3.47. Adolphson et al studied piroxicam for 8 weeks and found that none of the patients had nonunions, similar to control. Similarly, Drendel et al studied ibuprofen compared to acetaminophen, where both groups did not have any nonunions; however, the main purpose of the study was not to investigate bone healing, and, therefore, there was no strong evidence. Since these studies have zero events, the OR cannot be statistically calculated. Brattwall et al^[54]^ conducted an RCT that studied COX-2 inhibitors for a short time and found no significant difference of nonunions compared to control. Burd et al^[55]^ conducted an RCT that studied indomethacin for long duration and found a significant formation of nonunion. In contrast, Davis et al studied flurbiprofen for long duration and found no significant difference in bone healing.^[56]^ Lastly, Sagi et al^[58]^ also studied indomethacin for both short and long durations, finding that short duration did not show a significant difference, whereas long duration did.

These studies were subcategorized to have a better understanding of the type and duration of NSAIDs that had an influence on bone healing. After classifying the duration of the NSAIDs, it was found that short duration (less than 2 weeks) had an OR of 1.56 and was not significantly different, whereas long duration NSAIDs use had an OR of 5.27 and was significantly different compared to control. This means that short use of NSAIDs as a means of pain management might not have a significant effect on risk of nonunion formation. In contrast, long duration does have a significant increased risk of nonunion. This is an important factor that can influence the clinical management of patients.

A second subgroup that was studied was the type of NSAIDs used in the RCTs. Indomethacin, an acetic acid derivative, is a nonselective COX inhibitor. The studies that did not include indomethacin had an OR for nonunion of 2.58, which was not significant, whereas the studies that included only indomethacin had an OR of 3.87, which was significant. This may be a good indicator as to which NSAIDs to prescribe in order to decrease the risk of nonunion.

## 5 Limitations

The present meta-analysis does have its limitations. The studies that were included were limited to only 6 RCTs, which limit the analysis that is possible to have a better understanding of NSAIDs on bone healing. Among these studies, the primary outcome of nonunion was not standardized in terms of outcome measurement, some used X-ray or CT scan, whereas others used clinical evaluation for healing. For this reason, extraction of the data was limited to what was made available. Additionally, this meta-analysis did not consider the heterogeneity of the population and the targeted fracture sites that were chosen for the study. This would require a greater availability of studies. The studies that were included in the meta-analysis included various fracture types. For example, Adolphson, Davis, and Drendel included Colles and pediatric fractures of the upper extremities. Such fractures are uncommon to have nonunion; therefore, to equate the risk of nonunion of these fractures to, for example, long bones in adults would underestimate the number of possible nonunions. Additionally, the elective surgeries of hallus valgus described in Brattwal have a different healing potential compared to the high risk of nonunion in long bones. For these reasons, the limitation of the number of available RCTs on this topic has made the heterogeneity of the studies large.

## 6 Conclusions

Based on the available literature of NSAIDs exposure on bone healing, it can be concluded that there is a need for further studies to have a better understanding of the mechanism of action of different types of NSAIDs. From this meta-analysis, it can be concluded that NSAIDs that do not include indomethacin can be used for pain management without having a significant effect on bone healing and, additionally, the use of NSAIDs for short duration, less than 2 weeks, does not show a statistical increase in nonunions.
